# Evaluation of Student Pharmacists’ Attitudes and Perceptions of Hormonal Contraception Prescribing in Indiana

**DOI:** 10.3390/pharmacy9040185

**Published:** 2021-11-12

**Authors:** J. Henry Papineau, Jenny L. Newlon, Ryan S. Ades, Veronica Vernon, Tracey A. Wilkinson, Lynn M. Thoma, Ashley H. Meredith

**Affiliations:** 1 Pharmacy Department, HealthLinc, Valparaiso, IN 46383, USA; hpapineau@healthlincchc.org (J.H.P.); lthoma@healthlincchc.org (L.M.T.); 2 College of Pharmacy, Purdue University, West Lafayette, IN 47907, USA; jnewlon@purdue.edu; 3 College of Pharmacy, Manchester University, Fort Wayne, IN 46962, USA; RAdes@manchester.edu; 4 College of Pharmacy and Health Sciences, Butler University, Indianapolis, IN 46208, USA; vvernon@butler.edu; 5 School of Medicine, Indiana University, Indianapolis, IN 46202, USA; tracwilk@iu.edu

**Keywords:** hormonal contraceptive, Indiana, students, pharmacists, pharmaceutical services, counseling, attitudes, scope of practice

## Abstract

Community pharmacists’ scope of practice is expanding to include hormonal contraceptive prescribing. Prior to introducing statewide legislation, it is important to assess the perceptions of future pharmacists. A cross-sectional survey was distributed to 651 third- and fourth-year professional students enrolled at three colleges of pharmacy in Indiana. Data were collected between September and October 2019 to assess students’ attitudes about prescribing hormonal contraceptives, readiness to prescribe, perceived barriers, and desire for additional training. In total, 20.9% (n = 136) students responded. Most (89%, n = 121) believe that pharmacist-prescribed hormonal contraceptives would be beneficial to women in Indiana, and 91% (n = 124) reported interest in providing this service. Liability, personal beliefs, and religious beliefs were the most commonly cited perceived barriers. Most students felt they received adequate teaching on hormonal contraceptive methods (90%, n = 122) and hormonal contraceptive counseling (79%, n = 107); only 5% (n = 7) felt ready to provide the service at the time of survey completion. Student pharmacists in their final two years of pharmacy school are interested in prescribing hormonal contraceptives and believe that this service would be beneficial. This expansion of pharmacy practice would likely be supported by future pharmacists who feel the service could provide benefit to women seeking hormonal contraceptives in Indiana.

## 1. Introduction

Pharmacists are addressing important public health issues and increasing access to care by providing an increasing range of services. For example, pharmacists in several states, including Indiana, are now permitted to provide tobacco cessation products via a standing order [1]. This service improves patient access to smoking cessation products at their local community pharmacy, without requiring an in-office provider visit.

Pharmacists are also helping to address another important public health issue by prescribing hormonal contraceptives in several states—unintended pregnancies. According to the most recent data from 2011, almost half of pregnancies in Indiana are unintended (49%), which is above the national average of 45% [2,3,4]. In 95% of unintended pregnancies, women cite lack of or inconsistent use of hormonal contraceptives [2]. 

Although hormonal contraceptives can reduce the rates of unplanned pregnancy, barriers to access prevent some women from using hormonal contraceptives. One of the most commonly cited barriers is finding conveniently located providers [5]. Patients living in rural areas may lack access to prescribers to receive care and prescriptions for effective forms of birth control due to limited provider availability. However, nearly 90% of people within the United States live within 5 miles of a pharmacy, making pharmacists well-positioned to serve as a resource to provide access to and education on contraceptive methods [6]. Several states have leveraged pharmacists’ unique position within communities to increase access to contraceptive services through policies that allow pharmacists to select and dispense hormonal contraceptives for women. In these states, access via pharmacists has demonstrated that this service is effective in providing access to hormonal contraceptives in the community setting and can prevent unintended pregnancies while also saving states money related to pregnancy-related care [7,8]. Patients, pharmacists, and physicians have expressed interest in allowing pharmacists to prescribe hormonal contraceptives [9,10,11,12,13,14,15,16]. When surveyed, a majority of women said that they would utilize pharmacist-prescribed hormonal contraceptive services [10], and most pharmacists would be interested in providing such services [13,14]. Lastly, the American College of Obstetricians and Gynecologists recognizes pharmacist prescribing as an acceptable intermediate step towards transitioning hormonal contraceptives to over-the-counter status [17].

Pharmacists’ perceptions and motivation to prescribe hormonal contraceptives were not assessed prior to legislation to this effect being written and passed in Oregon and California. After the legislation passed in Oregon, only 39.1% of respondents planned to prescribe hormonal contraceptives [18] and most community pharmacists surveyed in California were not aware of the new legislation [19]. Even after implementation, 76.1% of pharmacists report writing fewer than 10 prescriptions for hormonal contraceptives per month [20]. While most respondents were supportive of the expansion of practice, there was a call for additional training and resources to make implementation of the new legislation effective [19]. Most pharmacists also cited time constraints, lack of reimbursement, and liability as barriers to implementing this service [21]. 

A bill allowing pharmacists to prescribe hormonal contraceptives is currently being considered in Indiana [22]. This bill seeks to allow pharmacist prescribing of pills and patches to people aged 18 years and older. Studies have demonstrated that student pharmacists were supportive of such expansion of practice before it became a reality in states such as California [23]. Although student pharmacist perceptions of hormonal contraception prescribing have been assessed in states that do not yet allow pharmacists to provide this service, such as North Carolina [24] and Alabama [25], it is important to understand how student pharmacists in Indiana close to graduation perceive prescribing hormonal contraceptives. Many student pharmacists graduating from pharmacy schools in Indiana will stay and practice in the state [26]. While preparing current pharmacists for expansion of service is critical, it is important to not overlook student pharmacists who will soon enter the workforce and, eventually, comprise a large portion of pharmacists in the state. Therefore, it is necessary to gauge Indiana student pharmacists’ perceptions and attitudes towards providing such services. The assessment of student pharmacists is crucial as their readiness to provide a new service can identify potential gaps in the current approaches to student training, allow for adaptations to better prepare future pharmacists, explore perceived barriers, and increase support for legislation expanding pharmacists’ scope of practice. 

The objective of this study was to assess the perceptions and attitudes of student pharmacists attending school in Indiana regarding pharmacist prescribing of hormonal contraceptives. The survey focuses on assessing third- and fourth-year student pharmacists’ perceptions and attitudes regarding interest in prescribing hormonal contraceptives, perceived benefit of allowing pharmacists to prescribe hormonal contraceptives in Indiana, training regarding hormonal contraceptives, comfort prescribing various hormonal contraceptive methods, and perceived barriers to implementation using a combination of yes/no questions, Likert scale responses, and open-ended questions (see Appendix A). 

## 2. Materials and Methods

### 2.1. Study Design

A cross-sectional, anonymous, online survey was distributed to Indiana student pharmacists enrolled at Purdue University (a public pharmacy school), Butler University, and Manchester University (non-public pharmacy schools). The 17-item survey was developed specifically for this project as no previously validated surveys were available that would meet the study objective. All pilot testing of the survey was conducted by the research team and it was estimated to take five minutes to complete. Demographic data, such as anticipated graduation year, were also collected to help identify trends in the data. The study was deemed exempt by the institutional review board at Purdue University.

### 2.2. Sample 

To be eligible for inclusion in the study, the respondent had to be a student pharmacist enrolled at one of Indiana’s three colleges of pharmacy during the 2019–2020 academic year, be at least 18 years of age, and able to read English.

### 2.3. Data Collection

Data were collected online via Qualtrics between September and October 2019. Survey distribution was managed by each institution and was distributed via direct email at Manchester University and through a weekly electronic newsletter at Purdue University and Butler University. One reminder email was sent to the students two weeks after the initial email inviting students to participate.

### 2.4. Data Analysis 

Chi-square tests were used to compare responses between genders, year in professional pharmacy program, institution type, intended career path, and age. Responses were compared between students interested in direct patient care in a community setting and those pursuing other pharmacy career paths. The direct patient care in a community-based setting included ambulatory care, hospital outpatient, community pharmacy chain, independent community pharmacy, or grocery store pharmacy settings. Those pursuing pharmacy career paths outside of the community setting in the hospital career-path included specialty ambulatory care, inpatient hospital, or “other”. The significance level was set at *p* < 0.05 a priori for all statistical tests. A post hoc analysis of third- and fourth-year student pharmacist responses was completed given the students’ proximity to entering practice. 

## 3. Results

A total of 651 third- and fourth-year student pharmacists enrolled at Butler, Manchester, and Purdue Universities during the 2019–2020 academic year were invited to participate in the study. There were 332 students set to graduate in 2021 (third-year students) and 319 set to graduate in 2020 (fourth-year students). A total of 136 (20.9% response rate) third- and fourth-year pharmacy students responded to the survey. Key demographic information of respondents can be found in Table 1.

The majority of students (91.2%, n = 124) were interested in prescribing hormonal contraceptives. In addition, the majority of respondents (90%, n = 122) believe this service would be beneficial to women in Indiana. No statistically significant differences between the compared demographic cohorts (e.g., community vs. hospital career interest, anticipated graduation year, gender, pharmacy school, etc.) were found for either perception.

### 3.1. Education

When asked about education received on specific contraceptive methods, 122 respondents (89.7%) felt that they had adequate teaching, 12 (8.8%) were “neutral”, and 2 (1.5%) did not feel they received adequate teaching. The majority of respondents (78.7%, n = 107) agreed that they received adequate education about hormonal contraceptive counseling, 16 (11.8%) were “neutral”, and 12 (8.8%) disagreed. While most students felt that they received adequate education about hormonal contraceptive methods and counseling, only 7 (5.2%) felt that they would be ready to provide this service at the time of completing the survey. Most respondents (88.9%) reported that they would prefer some form of additional training (i.e., online course, live training, other) to feel more comfortable prescribing hormonal contraceptives as pharmacists. The remaining eight respondents (5.9%) reported they would never be comfortable providing this service.

### 3.2. Comfort with Prescribing 

Student pharmacists were asked to rate how comfortable they were with prescribing different forms of hormonal contraceptives (Table 2). The majority of students felt extremely or somewhat comfortable with prescribing all forms of hormonal contraceptives. Students felt most comfortable with the idea of prescribing combined oral contraceptives (n = 112, 83%), progestin-only pills (n = 103, 76.3%), and emergency contraceptives (n = 115, 85.2%) and least comfortable with IUDs (n = 85, 63%), the injection (n = 93, 68.9%), and the transdermal patch (n = 96, 71.1%).

### 3.3. Concerns

Concerns with pharmacist hormonal contraceptive prescribing were identified by 14 (10.4%) respondents. Five (3.7%) cited liability concerns, two (1.5%) cited personal beliefs, two (1.5%) cited their religious beliefs, four (3%) cited concerns with pharmacist training, and one (0.7%) felt this service was unnecessary. Eleven (8.1%) students were not interested, while four (3%) students who believed the service to be beneficial were also not interested. Only student pharmacists indicating a lack of interest in prescribing hormonal contraception were prompted to identify their concerns. This revealed an error in the survey skip logic utilized as the intent was for all students to be able to respond to this item.

## 4. Discussion

Overall, the results demonstrate that the third- and fourth-year student pharmacists surveyed believe pharmacist prescribing of hormonal contraceptives would be beneficial to women in Indiana and they are interested in providing this service after graduation. In particular, the majority (n = 85/104, 81.7%) of female respondents had a history of using hormonal contraceptives and also believe this service would be beneficial for women in Indiana. These findings are similar to what Mospan et al. found among pharmacy students in North Carolina, with 80.4% of female respondents having a history of using hormonal contraceptives [24]. Although women in the study were not significantly more likely to support or oppose the delivery of this service than men, women’s personal experiences accessing, or attempting to access, hormonal contraceptives in Indiana may provide valuable insight into the benefits of allowing pharmacists to provide this service in Indiana.

Even though most respondents to the survey felt they received adequate education about hormonal contraceptive methods and counseling, the desire for additional training prior to prescribing hormonal contraceptives was consistently identified. Data from states that have implemented pharmacist hormonal contraceptive prescribing have also shown that licensed pharmacists felt they needed more training for implementation to be effective [19,20,21]. While the desire for additional training was apparent in several studies in addition to this study, a study aimed at student pharmacists in California did not find a perceived need for additional training [23]. Most of the California student pharmacists had received additional contraceptive training as part of required courses, and it is possible that this additional training or other variations in pharmacy curricula across states could account for the difference in comfort levels. With any new service, or expansion of service, it makes sense that students would feel more comfortable with additional training as it has been demonstrated that students feel more confident with more practice [27]. This is especially true since student pharmacists may not have observed provision of this service modeled by their preceptors during the experiential learning portion of their education or while working as a pharmacy intern. In line with this, Hohmann et al. found the most preferred learning method among student pharmacists in their study was to watch videos of pharmacists counseling patients on hormonal contraceptives [25]. Additionally, student pharmacists could benefit from more specific training on pharmacy service provision and prescribing of medications, including hormonal contraceptives, in their pharmacy school curriculum. Traditionally, many pharmacy curricula focus on characteristics of medications, such as mechanism of action, and guideline-recommended treatment regimens. Collaboration among faculty members across the state should be explored to lead to consistent training of student pharmacists in a manner that allows them to feel prepared and confident in prescribing hormonal contraceptives upon graduation.

For the success of pharmacist hormonal contraceptive prescribing in Indiana, it is vital to avoid implementation issues that have been encountered in other states, such as lack of awareness among pharmacists [19]. Awareness of the legal ability to provide a new service is the critical first step to engaging pharmacists to implement the service; therefore, it is necessary to understand what may be contributing to this lack of awareness. One possible cause of this lack of awareness is the failure to comprehensively assess pharmacists’ and student pharmacists’ perceptions before enacting policies allowing pharmacists to prescribe hormonal contraceptives. Ensuring that Indiana’s student pharmacists and pharmacists are planning to participate in the expansion of practice is vital for its widespread success [19,20,21]. For example, in August 2019, the Indiana State Department of Health issued a statewide standing order for smoking cessation, giving community pharmacists the ability to prescribe all FDA-approved smoking cessation products. While this initiative permits pharmacists to use more of their clinical expertise, we are unaware of any research into pharmacists’ and student pharmacists’ perceptions of the service. Many pharmacies in Indiana are not currently utilizing the smoking cessation standing order [28].

The findings can be used to inform legislation to make pharmacist-prescribed hormonal contraceptives a reality in Indiana. While Indiana student pharmacists are interested in prescribing hormonal contraceptives and believe it would be beneficial, they still identified potential barriers. Liability, religious/personal beliefs, and lack of training were the most common barriers cited in the study. These results are consistent with other surveys that cited similar barriers [19,20,21,23,24]. By adding prescribing of hormonal contraceptives as a focal point in their curriculum, some barriers cited by student pharmacists will likely be addressed.

One strength of this study was that the demographic characteristics of respondents with respect to race, ethnicity, gender, and age were similar to those of student pharmacists in the state of Indiana, making the results fairly representative of Indiana’s next generation of pharmacists. A potential limitation is that no sample size calculations were performed for subgroup comparisons, so it is possible that a significant difference may have been found with a larger sample. Due to survey distribution being managed at the individual pharmacy schools, methods varied slightly by institution, leading to variable response rates between institutions. Students at Manchester University received a direct email from their experiential education office with survey information, while students at Purdue University and Butler University were notified about the survey via a weekly electronic newsletter containing information about a variety of topics. Due to an error in survey skip logic identified after release of the survey, only a small number of respondents viewed the item assessing perceived barriers. For this reason, the study may not have adequately assessed the perceived barriers of students in the state of Indiana, which could result in potential resistance when implementing the service in the future; however, it is also possible that students would have a misperception of barriers due to potential lack of current experience in the community pharmacy setting.

## 5. Conclusions

The student pharmacists in Indiana surveyed believe expanding practice to include pharmacist prescribing of hormonal contraceptives would be beneficial for women in Indiana, and they are interested in providing this service. Despite perceived adequate training on hormonal contraceptive methods and counseling, student pharmacists would feel more comfortable providing this service with additional training. Regardless of age, gender, institution type, or anticipated career path, students were supportive of pharmacists gaining the legal ability to prescribe hormonal contraceptives. Future assessment of pharmacists, student pharmacists, and other healthcare professionals may be necessary to further strengthen the successful implementation of this expansion of pharmacy practice.

## Figures and Tables

**Table 1:** Demographic characteristics of participants (N = 136).

	n	%
Age		
20–24 years old	105	77.2%
25–34 years old	26	19.1%
35–44 years old	5	3.7%
Race		
White	121	89.0%
Non-white	15	11.0%
Ethnicity		
Hispanic or Latino	4	2.9%
Non-Hispanic or Latino	132	97.1%
Gender		
Men	30	22.1%
Women	105	77.2%
Other	1	0.7%
Year in Pharmacy School		
Third year	56	41.2%
Fourth year	80	58.8%
Pharmacy School		
Public	25	18.4%
Non-public	111	81.6%
Career Interest		
Community ^a^	71	52.2%
Hospital ^b^	65	47.8%
Personal Use of Hormonal Contraceptives ^c^		
Used before	85	81.7%
Never used	19	18.3%
Prefer not to answer	0	0.0%

^a^ Ambulatory care, hospital outpatient, chain community pharmacy, independent community pharmacy, and grocery store community pharmacy. ^b^ Specialty ambulatory care, inpatient, other. ^c^ Only respondents identifying as a woman were presented with this question.

**Table 2:** Comfort with prescribing different forms of hormonal contraceptives.

	Extremely Uncomfortable		Somewhat Uncomfortable		Neither Comfortable nor Uncomfortable		Somewhat Comfortable		Extremely Comfortable	
**Combined Oral Pill**	**n**	**%**	**n**	**%**	**n**	**%**	**n**	**%**	**n**	**%**
All respondents	4	3.0%	12	8.9%	7	5.7%	48	35.6%	64	47.4%
Third-year students	3	5.5%	2	3.6%	1	1.8%	18	32.7%	31	56.4%
Fourth-year students	1	1.3%	10	12.5%	6	7.5%	30	37.5%	33	41.3%
Community	1	1.4%	6	8.6%	3	4.3%	24	34.3%	36	51.4%
Hospital	3	4.6%	6	9.2%	4	6.2%	24	36.9%	28	43.1%
**Progestin-only Pill**										
All respondents	4	3.0%	14	10.4%	14	10.4%	48	35.6%	55	40.7%
Third-year students	3	5.5%	3	5.5%	2	3.6%	20	36.4%	27	49.1%
Fourth-year students	1	1.3%	11	13.8%	12	15.0%	28	35.0%	28	35.0%
Community	1	1.4%	6	8.6%	9	12.9%	25	35.7%	29	41.4%
Hospital	3	4.6%	8	12.3%	5	7.7%	23	35.4%	26	40%
**Transdermal Patch**										
All respondents	5	3.7%	17	12.6%	17	12.6%	50	37.0%	46	34.1%
Third-year students	3	5.5%	5	9.1%	1	1.8%	25	45.5%	21	38.2%
Fourth-year students	2	2.5%	12	15.0%	16	20.0%	25	31.3%	25	31.3%
Community	1	1.4%	6	8.6%	12	17.1%	29	41.4%	22	31.4%
Hospital	4	6.2%	11	16.9%	5	7.7%	21	32.3%	24	36.9%
**Injection**										
All respondents	5	3.7%	21	15.6%	16	11.9%	50	37.0%	43	31.9%
Third-year students	4	7.3%	4	7.3%	3	5.5%	22	40.0%	22	40.0%
Fourth-year students	1	1.3%	17	21.3%	13	16.3%	28	35.0%	21	26.3%
Community	1	1.4%	9	12.9%	9	12.9%	25	35.7%	26	37.1%
Hospital	4	6.2%	12	18.5%	7	10.8%	25	38.5%	17	26.2%
**Intravaginal Ring**										
All respondents	4	3.0%	17	12.6%	8	5.9%	56	41.5%	50	37.0%
Third-year students	3	5.5%	4	7.3%	1	1.8%	21	38.2%	26	47.3%
Fourth-year students	1	1.3%	13	16.3%	7	8.8%	35	43.8%	24	30.0%
Community	1	1.4%	8	11.4%	5	7.1%	27	38.6%	29	41.4%
Hospital	3	4.6%	9	13.8%	3	4.6%	29	44.6%	21	32.3%
**Intrauterine Device**										
All respondents	7	5.2%	28	20.7%	15	11.1%	43	31.9%	42	31.1%
Third-year students	4	7.3%	7	12.7%	5	9.1%	13	23.6%	26	47.3%
Fourth-year students	3	3.8%	21	26.3%	10	12.5%	30	37.5%	16	20.0%
Community	3	4.3%	13	18.6%	7	10%	23	32.9%	24	34.3%
Hospital	4	6.2%	15	23.1%	8	12.3%	20	30.8%	18	27.7%
**Emergency Contraception**										
All respondents	7	5.2%	8	5.9%	5	3.7%	46	34.1%	69	51.5%
Third-year students	4	7.3%	2	3.6%	1	1.8%	17	30.9%	31	56.4%
Fourth-year students	3	3.8%	6	7.5%	4	5.0%	29	36.3%	38	47.5%
Community	3	4.3%	3	4.3%	2	2.9%	25	35.7%	37	52.9%
Hospital	4	6.2%	5	7.8%	3	4.6%	21	32.3%	32	49.2%

## Data Availability

Not applicable.

## Appendix A

Survey

Recently, the scope of practice has grown tremendously for pharmacists as an answer to increasing access to healthcare. A few states including Oregon, Colorado, and Idaho have passed laws allowing pharmacists to prescribe birth control, as well as some other medications. This survey aims to assess the utility of this practice for Indiana pharmacists. The survey consists of roughly 15 questions and should take no longer than 5 min. to complete. We value your honest feedback as we aim to advance pharmacy practice for Indiana pharmacists!

1. What is your age?

< 19 years old

20–24 years old

25–34 years old

35–44 years old

45–54 years old

> 55 years old

2. What is your race?

White

Black or African American

American Indian or Alaska Native

Asian

Native Hawaiian or Pacific Islander

Multiple

Other

3. What is your ethnicity?

Hispanic or Latino

Non-Hispanic or Latino

4. How would you describe yourself?

Male

Female

Non-Binary/Third Gender

Transgender Male

Transgender Female

Gender Variant/Non-Conforming

Prefer Not to Answer

5. In what year do you expect to receive your highest pharmacy degree (terminal degree)?

2020

2021

2022

2023

2024 or later

6. From what type of institution do you expect to receive your highest pharmacy degree (terminal degree)?

Public

Private, non-religious

Private, religious

Not sure

7. How would you best describe the pharmacy setting in which you are planning to practice the majority of the time?

Community pharmacy (chain, grocery)

Community pharmacy (independent)

Ambulatory care (primary care)

Ambulatory care (specialty care)

Hospital inpatient pharmacy (dispensing)

Hospital outpatient pharmacy

Hospital clinical pharmacy (inpatient care)

Other (fill in the blank)

8. How much do you agree with the following statement: “My pharmacy education included adequate teaching on contraception methods.”

Strongly Agree

Agree

Neutral

Disagree

Strongly Disagree

I have not yet been taught this material

9. How much do you agree with the following statement: “My pharmacy education included adequate teaching on contraception counseling.”

Strongly Agree

Agree

Neutral

Disagree

Strongly Disagree

I have not yet been taught this material

10. Other states such as Oregon, West Virginia, and Utah have expanded the scope of practice for pharmacists to include prescribing of contraception to women. Patients must complete a questionnaire regarding contraindications to hormonal contraceptive use and possible pregnancy status. Blood pressure is checked and certain counseling points are required. Do you believe a service like this would be beneficial in Indiana?

Yes

No

11. Why do you feel that pharmacist contraception prescribing would **not** be beneficial in Indiana? Select all that apply. (*Respondents only saw this question if they answered “No” to the previous question)

Personal beliefs (i.e., “I don’t believe pharmacists should prescribe medications”)

Religious beliefs (i.e., “My religion does not support use of birth control”)

Training (i.e., “Pharmacists do not have the proper training to prescribe contraceptives”)

Unnecessary (i.e., “Women in Indiana have adequate access to contraception already”)

Liability (i.e., “I don’t want to be responsible for prescribing birth control. ”)

12. In an ideal setting where the scope of practice allows, and you had all of the necessary time, resources, training, and reimbursement, would you be interested in prescribing hormonal contraception?”

Yes

No

13. If no, why are you not interested in prescribing hormonal contraceptives? (select all that apply) (*Respondents only saw this question if they answered “No” to the previous question)

Personal beliefs (i.e., “I don’t believe pharmacists should prescribe medications”)

Religious beliefs (i.e., “My religion does not support use of birth control”)

Liability concerns (i.e., “If a patient experiences a bad reaction, I will be held professionally and financially responsible”)

Fear of women neglecting recommended health care (i.e., “If I prescribe contraceptives, the patient may not visit her provider for a pap smear or other screening”)

Comfort level (i.e., “I am not comfortable discussing birth control with other people”)

Employer (i.e., My employer does not allow prescribing of birth control”)

Knowledge (i.e., “I need additional education to provide this service”)

Workflow (i.e., “I am too busy to add additional services with the current staffing”)

Environment (i.e., “I do not have a private counseling room to provide this service”)

Access to patient information (i.e., “I don’t have access to patient medical records for adequate history/screening purposes”)

Other (fill in the blank)

14. If allowed within the scope of practice for pharmacists in Indiana, how comfortable would you be prescribing and counselling on the following hormonal contraceptives?

	**Extremely** **Comfortable**	**Somewhat** **Comfortable**	**Neither Comfortable nor Uncomfortable**	**Somewhat** **Comfortable**	**Extremely** **Uncomfortable**
Combined (estrogen/ progestin) oral pill					
Progestin only oral pill					
Transdermal (i.e., Xulane)					
Injection (i.e., Depo-Provera)					
Intravaginal (i.e., NuvaRing)					
Intrauterine device (i.e., Mirena, Paragard)					
Emergency contraception pill (i.e., ella, PlanB)					

15. What additional training would be necessary for you to prescribe contraception comfortably, if allowed within the scope for practice for pharmacists in Indiana?

Online course (3–4 h)

Online course (1–2 h)

Live training (3–4 h)

Live training (1–2 h)

No additional training necessary—I feel comfortable to provide this service right now

No amount of additional training would make me comfortable

I don’t know, I have not been taught this material yet in school

Other (fill in the blank)

16. Have you personally ever used hormonal contraception (i.e., pills, patch, vaginal ring, depo, IUD, implant)? (*Only respondents selecting that they identify as “Female” saw this question)

Yes

No

Prefer not to answer

17. Have you personally ever experienced problems accessing hormonal contraception? (*Only respondents selecting that they identify as “Female” saw this question)

Yes

No

Prefer not to answer
